# An Efficient Scalable Scheduling MAC Protocol for Underwater Sensor Networks †

**DOI:** 10.3390/s18092806

**Published:** 2018-08-25

**Authors:** Faisal Alfouzan, Alireza Shahrabi, Seyed Mohammad Ghoreyshi, Tuleen Boutaleb

**Affiliations:** School of Engineering and Built Environment, Glasgow Caledonian University, Lanarkshire G4 0BA, Glasgow, UK; A.Shahrabi@gcu.ac.uk (A.S.); Seyed.MohammadGhoreyshi@gcu.ac.uk (S.M.G.); T.Boutaleb@gcu.ac.uk (T.B.)

**Keywords:** underwater sensor networks, media access control, collision-free MAC protocols, duty cycle mechanism, depth-based scheduling

## Abstract

Underwater Sensor Networks (UWSNs) utilise acoustic waves with comparatively lower loss and longer range than those of electromagnetic waves. However, energy remains a challenging issue in addition to long latency, high bit error rate, and limited bandwidth. Thus, collision and retransmission should be efficiently handled at Medium Access Control (MAC) layer in order to reduce the energy cost and also to improve the throughput and fairness across the network. In this paper, we propose a new reservation-based distributed MAC protocol called ED-MAC, which employs a duty cycle mechanism to address the spatial-temporal uncertainty and the hidden node problem to effectively avoid collisions and retransmissions. ED-MAC is a conflict-free protocol, where each sensor schedules itself independently using local information. Hence, ED-MAC can guarantee conflict-free transmissions and receptions of data packets. Compared with other conflict-free MAC protocols, ED-MAC is distributed and more reliable, i.e., it schedules according to the priority of sensor nodes which based on their depth in the network. We then evaluate design choices and protocol performance through extensive simulation to study the load effects and network scalability in each protocol. The results show that ED-MAC outperforms the contention-based MAC protocols and achieves a significant improvement in terms of successful delivery ratio, throughput, energy consumption, and fairness under varying offered traffic and number of nodes.

## 1. Introduction

Underwater sensor networks (UWSNs) have a promising future in the area of information collection with increasingly more applications in recent years. These applications vary from environmental monitoring and oceanographic data collection to early warning systems, tactical surveillance, assisted navigation, and resource discovery [1,2,3,4].

Similar to other ad hoc networks, a UWSN consists of a number of sensors that are deployed in water to collaboratively perform their tasks over a given area [5]. Unlike terrestrial sensor nodes that rely on radio signals to communicate with each other, the nodes use the acoustic channel as the communication medium with 1500 m/s speed, which is five orders of magnitude lower than that of radio signals. As a result of the lower propagation speed, higher propagation delays occur in communication [6,7]. In addition, the available bandwidth of acoustic channels is typically less than 15 kHz, which is much narrower compared with that of terrestrial channels. Apart from long propagation delay and narrow bandwidth, energy-efficiency is another primary concern due to difficulties of replacing or recharging batteries. Furthermore, UWSNs usually cover a very large area of the ocean environment, which leads to sparse deployment [8,9]. Due to these characteristics of underwater environment, the design of Medium Access Control (MAC) protocols has become a challenging task [10,11].

The most critical challenge of underwater MAC protocols is perhaps the spatial-temporal uncertainty. In UWSNs, uncertainty in the receiving time which depends on the transmitting time (time uncertainty) as well as the propagation delay to the destination (space uncertainty) [8]. Thus, transmissions time and location (e.g., propagation delay) of sensors should be considered in order to make a collision-free scheduling of transmissions and receptions. Moreover, a collision can also be caused by a hidden terminal. This case occurs when a sensor node cannot sense other nodes that interfere with its transmission. To enable collision-free scheduling of transmissions and receptions, these problems should properly be addressed.

To solve these issues, some handshake-based MAC protocols have been proposed [6,12]. In this approach, a Request-To-Send/Clear-To-Send (RTS/CTS) mechanism is used to reserve the channel. Floor Acquisition Multiple Access (FAMA) [13] lengthens the transmission delays of RTS/CTS control packets to let Multiple Access with Collision Avoidance (MACA) [14] operate in the networks with long propagation delay such that in UWSNs. However, it consumes high energy by transmitting such long control packets. To reduce the energy consumption, S-FAMA [12] introduces a slot technique to FAMA by employing short RTS/CTS control packets while collision in UWSNs is still avoided. For the RTS/CTS mechanism, a one-time process usually reserves the channel for only one sender-receiver pair. Although energy efficiency is extremely improved in this way, the network throughput is usually low because of the high delay in the handshaking phase.

To improve the network throughput, random access-based MAC protocols, such as ALOHA and Slotted ALOHA, have been explored [15]. To achieve better performance, several random access-based MAC protocols are particularly designed for UWSNs, such as Aloha with Advance Notification (ALOHA-AN) [16] and UWAN-MAC [17], which employ short control packets without coordination. It means that when a data packet arrives at a receiver, if the receiver is not receiving any other packets and there is no other packet coming in this period, the packet transmission is successful. Thus, collision avoidance is totally probabilistic because they cannot handle the hidden terminal problems [9].

While the above-mentioned classes of MAC protocols are expected to achieve high performance, some recent observations, however, have reported that long propagation delay, narrow bandwidth, and high bit error rate make the contention-based MAC protocols costly [18,19,20]. Therefore, both handshake-based and random access-based MAC protocols are not as efficient as expected [19,20,21].

In contrast, contention-free MAC protocols are able to achieve higher level of efficiency. Most of these MAC protocols utilise a centralised scheduling method which spends a long time to globally collect the topology specifications from all sensors because of long propagation delay. As a result, a distributed scheduling method is preferred.

In this paper, we propose an Efficient Depth-based MAC protocol (ED-MAC) which takes the energy efficiency, throughput, fairness, and collision avoidance into consideration. ED-MAC schedules the transmissions and receptions of data packets at both the sender and receiver sides to not only achieve the above-mentioned objectives, but also to address the spatial-temporal uncertainty and hidden terminal problems. After scheduling the media access following a distributed manner, and the normal operational phase, each node is occasionally awake to either transmit or receive data according to its schedule. The nodes can sleep when there is no packet to transmit or receive, to save more energy.

The remainder of the paper is organised as follows: Section 2 introduces the related works. Section 3 presents challenges and requirements. Section 4 illustrates problem definition. Section 5 describes ED-MAC protocol in detail as well as discusses the offered traffic upper-bound and then analyses the number of slots of our proposed protocol. Section 6 presents the performance of ED-MAC protocol and compares it with UWAN-MAC and T-Lohi protocols through simulations. Finally, Section 7 concludes the paper.

## 2. Related Work

Packet retransmission is the major source of energy waste, i.e., sending a packet twice, leads to increased energy consumption [22]. Many underwater MAC protocols have recently emerged to overcome this and to cope with the features of underwater acoustic communications. These protocols can be classified into two categories: contention-free and contention-based [23,24,25].

In the contention-free category, communication channels are separated into time, frequency or code domains, such as Time Division Multiple Access (TDMA), Frequency Division Multiple Access (FDMA), and Code Division Multiple Access (CDMA) [26]. In TDMA, the channel is divided into a number of time slots. All sensor nodes have to remain synchronised to keep reliable transmission schedules by incurring additional control and scheduling packets [27]. UnderWater FLASHR (UW-FLASHR) [28], Spatial-Temporal MAC (ST-MAC) protocol [5], and Staggered TDMA Underwater MAC Protocol (STUMP) [29] are typical TDMA-based protocols for UWSNs. Some other TDMA-like approaches have also been proposed [30,31]. FDMA divides the frequency band into sub-bands. However, the narrow band of an acoustic channel leads to a low throughput because of the diffuse fading in underwater areas. CDMA is robust against frequency fading and able to improve the network throughput. Thus, receivers can distinguish between signals concurrently transmitted by multiple sensor nodes, which increases channel utilisation and decreases the packet retransmissions. Nevertheless, CDMA is not appropriate for UWSNs because it is difficult to set pseudo-random codes to many sensor nodes.

In contrast, the contention-based category can be more manageable and effective with dynamic network topologies and therefore more suitable for UWSNs. This type of protocol can be further classified into two groups, namely handshake-based and random access-based MAC protocols [9,25,32]. Protocols in different groups have notable performance on throughput, delay or energy efficiency. In the first group, handshaking-based, source and destination nodes exchange control packets before sending data packets, attempting to avoid any possible collision. In the second group, random access-based, nodes randomly attempt to access the medium. When a data packet arrives at the destination node, if there is no packet coming from other nodes, this node can receive the packet successfully. However, most of the efforts of the MAC protocol design for UWSNs have focused on the handshaking group such as S-FAMA [12], DACAP [33], DOTS [34], and R-MAC [6]. Some variances based on the handshaking group have also been proposed [35,36].

Slotted Floor Acquisition Multiple Access (S-FAMA) [12] is a handshaking-based protocol, which combines both carrier sensing (CS) and an initiate phase between the source and destination before data transmission. During the initiate phase, RTS/CTS control packets are exchanged between the source and the destination nodes to avoid two or more simultaneous transmissions. This means that S-FAMA uses these kinds of packets (e.g., RTS, CTS, DATA and ACK) before the data packets, which should also be sent at the beginning of a single slot, to schedule sender and receiver sides properly. Despite S-FAMA avoiding data packet conflicts without relying on the size of the packet, it needs a clock synchronisation between sensor nodes, which is difficult to obtain in UWSNs. Moreover, S-FAMA consumes more energy because of idle listening and overhearing.

In [33], a Distance-Aware Collision Avoidance Protocol (DACAP) is another handshaking-based protocol, which also merges CS and exchanging RTS/CTS control packets before sending data packets. However, it does not need any concurrence between nodes. Nevertheless, exchanging such control packets, between the source and destination to eliminate data packet collisions, consumes a significant amount of energy in UWSNs. Delay-aware Opportunistic Transmission Scheduling (DOTS) [34] is another handshaking-based approach, which utilises the network topology information and the handshaking mechanism to improve the protocol’s performance.

R-MAC [6] is another handshaking-based method mainly designed for energy efficiency and fairness. In this technique, packet collision is entirely avoided by accurately scheduling the transmissions of control and data packets. R-MAC not only avoids data packet collision, but can solve the exposed terminal problem inherited from the RTS/CTS-based protocols. By solving this problem, it can ideally save more energy together with supporting the fairness. In this protocol, instead of using RTS/CTS exchange control packets to avoid data packets’ collisions, the transmission of these packets is scheduled between both sender and receiver sides [37]. Moreover, to reduce energy consumption in the idle state and overhearing, every node operates in the listen and sleep modes periodically [2,8]. R-MAC trades off high end-to-end delay for energy efficiency and fairness, hence collision avoidance is achieved, but, when the offered traffic increases, only several data packets are transmitted [6].

Accordingly, the random access-based group usually allows sender nodes to transmit packets randomly or after an initial one-way contention [38]. UW-ALOHA [39] is a typical random access-based protocol for UWSNs. The unique features are the automatic repeat request (ARQ) and back-off schemes, which are used to improve the performance of traditional ALOHA in underwater environments. In [16] two enhanced designs for ALOHA in UWSNs are proposed. In ALOHA collision avoidance (ALOHA-CA) protocol, every packet is segmented into two separate parts: a header segment and a data segment. In this model, a sensor node can extract both transmitter and receiver information through a short overhearing. ALOHA with advanced notification (ALOHA-AN) is another random access-based approach, which includes sending a short data packet before the actual data transmission within the information of both sender and receiver [40]. A similar approach called T-Lohi is proposed in [41], which employs a tone-based contention mechanism to detect collisions. Technically, it uses the tone signal, which is short enough to avoid collisions.

UWAN-MAC [17] is also a random access-based process, which leverages local synchronisation to determine the time-line of each sensor node for energy efficiency improvement. It provides a suitable method by extending the sleep mode rather than the idle listening mode. This is because the former consumes less energy than that in the latter. In particular, UWAN-MAC is an energy efficient MAC protocol designed for UWSN. For example, when the source node transmits a packet, it informs the destination node when it is assumed to transmit the next packet; then, the neighbourhoods will overhear the packet to prevent any possible collisions [42]. The main issue with this technique is that the spatial-temporal uncertainty and the hidden terminal problem are not properly detected; therefore, it consumes significantly more energy because of the collisions and retransmissions.

## 3. Challenges and Requirements

In this section, we first identify the impact of long propagation delays and limited bandwidth for underwater networks. Secondly, the difficulties of replacing or recharging batteries in underwater networks mean that energy efficiency is a major concern, thus saving energy is explored. We finally describe our motivation for proposing an efficient depth-based MAC protocol.

### 3.1. Impact of Long and Variable Propagation Delays

**Figure 1:**
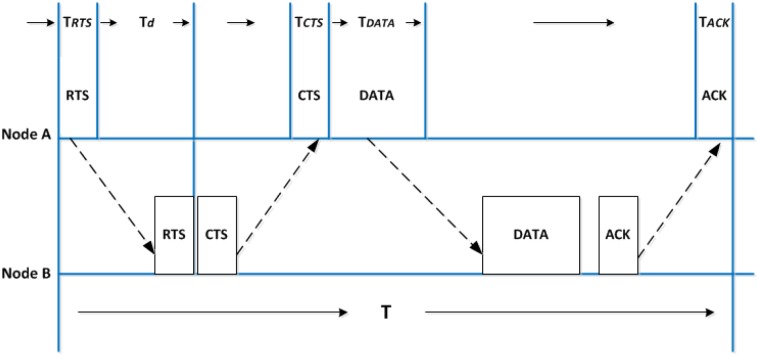
Handshake in terrestrial networks.

Long propagation delays in underwater acoustic channels mean that choosing the right MAC protocol design is critical [43]. Traditional mechanisms for collision detection, such as carrier sense, are no longer effective. The fundamental approach of using Carrier Sense Multiple Access with Collision Avoidance (CSMA/CA), for instance, is to avoid sending data in a busy channel, i.e., a node first listens to the channel before transmitting data. If the channel is idle, the data packet can be transmitted [44]. In underwater acoustic networks, however, due to long propagation delays, even when a node overhears a signal, it does not mean that its neighbours are experiencing the same thing at the same moment. The whole idea of detecting collision by listening to the channel is therefore not valid in this scenario. The performance of some advanced collision detection mechanisms for terrestrial wireless networks, however, such as the RTS/CTS handshake-based approach, is very low in underwater acoustic networks. Figure 1 shows the basic workflow of the classic RTS/CTS approach.

When node A wants to communicate with its neighbouring node B, it first transmits an RTS message (denoted as RTS in the diagram). Once B receives the message, it responses with a CTS message to confirm to the sender and triggers data transmission. The CTS message also informs other neighbours to avoid any potential collisions. After node A receives the CTS message, it starts delivering its data packet. After receiving the DATA packet, node B sends an ACK to node A to conclude the communication process.

Assuming the time spent on RTS/CTS/DATA/ACK are denoted as $T_{RTS}$, $T_{CTS}$, $T_{DATA}$, and $T_{ACK},$ respectively. Then, $T_{d}$ is used to denote propagation delay, while $T_{\epsilon }$ denotes the total system delay when switching between sending and receiving. The total time spent on transmitting one packet in Figure 1 is thus *T* = $T_{RTS}$ + $T_{CTS}$ + $T_{ACK}$ + $T_{DATA}$ + $T_{\epsilon }$ + 4 × $T_{d}$, without considering any collisions. Thereafter, the system performance, in terms of channel utilisation, is determined as [42]:

(1) $$\eta \leq \frac{T_{DATA}}{T}\leq \frac{T_{DATA}}{T_{DATA}+4\times T_{d}}=\frac{1}{1+4\times \alpha },$$

where α is known as the ratio of the propagation delay $T_{d}$ to transmission delay $T_{DATA}$, assuming that there are no collisions or link errors. For Radio Frequency (RF) wireless networks, α approaches 0, yielding high η. In UWSNs, however, α is much greater and non-negligible. When the $T_{d}$ controls the transmission time, which means that α is higher than 1, the channel utilisation becomes low [45].

In UWSNs, the propagation delay is highly variable, and depends on the depth of the water and the temperature [9]. Moreover, the temperature and salinity may vary over time which can affect the speed of sound as well. These features impact implications for the MAC protocol design. Nevertheless, variability in water speed, temperature, and salinity are out of scope of this paper. Some acoustic transducers cannot also transmit and receive in an omnidirectional way; however, the acoustic transducers are assumed to be omnidirectional in our proposed model.

### 3.2. Impacts of Low Bit Rate and Limited Bandwidth

The available acoustic bandwidth depends on the transmission distance, due to high environmental noise at low-medium frequencies (lower than 1 kHz or high-power absorption at high frequencies, which can be greater than 50 kHz [46]). Typically, acoustic modems work at frequencies from only a few Hz to tens of kHz. The bit rate in underwater sensor networks can therefore barely exceed 100 kbps. Technically, the limited bandwidth of acoustic channels requires the accurate design of coding schemes and MAC protocols for use in UWSNs.

### 3.3. Energy Consumption

In underwater acoustic networks, the transceivers have transmission powers an order of magnitude higher than that of the terrestrial networks, with a higher ratio of transmit-to-receive power. Therefore, protocols using the acoustic waves efficiently become much more critical in UWSNs [47]. Furthermore, saving energy is a major concern due to the difficulties of replacing or recharging batteries in underwater environments.

### 3.4. Motivation

Performance issues caused by long propagation delays, limited bandwidth, and energy consumption essentially result in the inefficiency of the handshake mechanism in acoustic channels. The handshaking process in underwater networks only allows for one data transmission in one control message, such as RTS/CTS, in the one-hop neighbourhood. If every node could be scheduled to transmit data at certain times with only one beacon window and one round window, the energy efficiency and throughput ratio would significantly improve. This is the basic driving idea of our proposed MAC protocol, in which spatial reuse (concurrent sending in different neighbourhoods) is introduced as a similar concept and has been developed based on acoustic channels for underwater networks.

To further improve the MAC performance, we consider the underwater features such as three-dimensionality and depth of sensors in our proposed protocol, as will be explained in detail later. We also employ the time division to access the channel and make some improvements on network schedule for common TDMA. In this case, the time slot assignment method is used for improving the throughput, energy efficiency, and fairness by imposing less communication overhead in the scheduling process compared to those other TDMA approaches, which are impractical in UWSNs.

## 4. Problem Definition

To achieve higher energy efficiency and network throughput, we target to design a collision-free MAC protocol while also addressing the following problems: hidden terminal and spatial-temporal uncertainty, which are described as follows:

### 4.1. Hidden Terminal Problem

**Figure 2:**
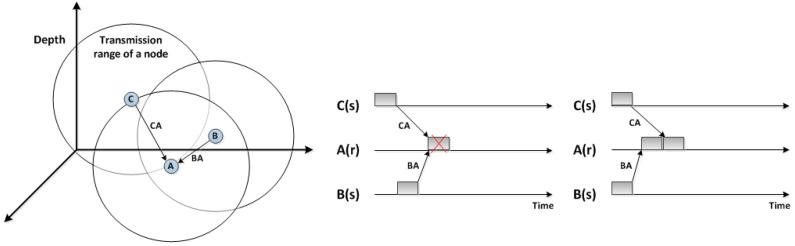
Impact of the hidden terminal problem and the long propagation delay on underwater MAC protocols.

The phenomenon of a hidden terminal occurs when a sensor node cannot sense one or more nodes that can overlap with its transmission. Figure 2 illustrates this situation in which sensors B and C are hidden from each other. Hence, a concurrent transmission by sensors B and C results in a collision in sensor A. The existence of a hidden terminal leads to increasing the number of collisions and retransmissions, and thus results in higher energy consumption and lower network throughput.

### 4.2. Spatial-Temporal Uncertainty Problem

To accurately solve the collision issue in terrestrial wireless networks and due to its short propagation delay, it is sufficient to limit the interfering sensor nodes from sending concurrently. In UWSNs, however, it is necessary to consider the transmission time and the location of the node because of the long propagation delay of acoustic waves. The spatial-temporal uncertainty problem can be described as a ’two-dimensional uncertainty’, which is defined as follows:

- The collision in the destination node is dependent on the propagation delay and transmission time; thus, it can be shown as a duality that differs between both the transmission time and the location of the sensor nodes.
- The distance between sensor nodes changes based on the uncertainty of current channel status and a data packet may collide even if no other nodes transmit concurrently.

Figure 2 shows how the high propagation delay in UWSNs could make a spatial-temporal uncertainty problem. This figure illustrates two cases of the spatial-temporal uncertainty problem. Firstly, when nodes B and C transmit packets with varying times of transmission, a collision might occur at node A. In the second case, when both nodes B and C start transmitting to node A simultaneously, there is no collision as their packets arrive at node A at different times. This is mainly because of various propagation delays.

## 5. Efficient Depth-Based MAC Protocol

In this section, we first brief the basic ideas of ED-MAC followed by describing each operational phase in detail. We next discuss how ED-MAC handles newcomer nodes, and finally analyse the offered traffic upper-bound and also the minimum number of slots required in our proposed protocol.

### 5.1. Overview of ED-MAC Protocol

Efficient Depth-based (ED-MAC) is a reservation-based MAC protocol. It employs a duty cycle mechanism by assigning time slots to every individual node in the network in a distributed manner. The primary goal is to reduce the energy consumption by using a wakeup scheduling scheme; nodes are awake in some slots to transmit or receive data and are asleep over the remaining slots. The possibility of collision is very slim and limited to a very specific scenario. To remove even this slight chance, every slot is divided into a number of sub-slots. These sub-slots are selected randomly to avoid collision across the network. The sub-slots are also used to handle the newcomer nodes. ED-MAC trades off latency for energy efficiency and fairness and hence provides the flexibility to be utilised for various energy-critical applications. A summary of the notations used to describe our model is given in Table 1.

**Figure 3:**
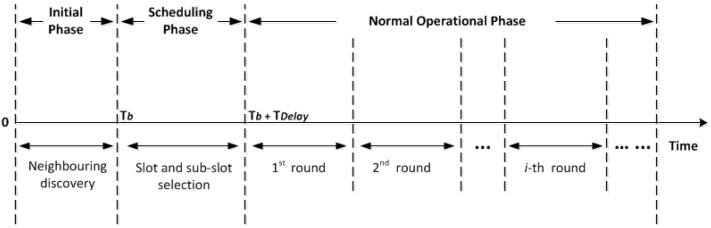
Timeline of ED-MAC protocol.

Nodes in the network operate in three phases; namely initial, scheduling, and normal operational phase, as depicted in Figure 3. All network nodes operate asynchronously during each phase but share a common clock to start and end each phase together. To eliminate the effect of any clock drift that may occur over a long period of time, a guard time is also applied.

In the initial phase, all the nodes randomly broadcast a few small beacons to discover their one-hop neighbouring nodes. The length of this phase, $T_{b}$, is a predefined fixed value for all nodes, which is set at each node before deployment.

The goal of the second phase, the scheduling phase, is to assign a unique slot to every node in the network. A timer is used at each node to prioritise slot reservation depending on the node depth; the greater the depth, the higher priority to reserve a slot. The length of the scheduling phase, $T_{Delay}$, should be long enough to provide all nodes, from the seabed to water surface, with the opportunity to reserve a slot for themselves, but it is significantly shorter than that of the third phase. It should be noted that the initial and the scheduling phases only require to be repeated when the network topology changes. However, handling newcomers can allow ED-MAC to repeat these phases less frequently.

The normal operational phase is divided into a number of rounds and each round is consisted of a number of slots. At each round, every node is aware of its own reserved slots and also the slots reserved by its neighbouring nodes. Therefore, they can schedule to wakeup either to transmit their own data packet during the reserved slots or to possibly receive a data packet from a neighbouring node. They are asleep in other remaining slots when there is no data transmission or reception. This pattern is repeated during every round. The length of this phase, i.e., the number of rounds, depends on the topology changes due to node displacement or energy depletion. A shorter length should be considered for this phase in those scenarios with rapid topology changes and a longer period in scenarios with stationary or limited mobile nodes. Either way, it is a predefined fixed value configured on each node before deployment.

### 5.2. Initial Phase

At the deployment time, the start time of the initial phase for each node is set. During the initial process, every node randomly broadcasts its beacon packet to its neighbourhood. The beacon packet includes the ID and depth of the sender. Each node exploits a pressure gauge, which is embedded inside the sensor, to obtain its depth [48]. The pressure gauge may give some variance in the depth reading; however, considering such an error in depth reading is out of scope of this work.

The purpose of this phase is to exchange the ID and depth between neighbouring nodes which can be used at each node to populate neighbouring table, $N_{t}$. Upon receiving a beacon packet, every node immediately updates its $N_{t}$. The length of this phase, $T_{b}$, is set to a predefined fixed value for all nodes. It is also a function of the transmission range of each node, $R_{tr}$, and the maximum number of nodes per neighbourhood, $N_{max}$, which should be long enough to let them create their own one-hop neighbouring tables with accurate information. However, the length of this phase is very short compared to that of the third phase.

### 5.3. Scheduling Phase

Using the information stored in the neighbouring table, $N_{t}$, during the first phase, every node in the network needs to reserve a unique slot for itself to use during the third phase for data transmission. It needs to know which slots are reserved by its one-hop neighbouring nodes to adjust its wakeup time as well. Hence, the goal of the second phase is to schedule the wakeup and sleep times at each node. A depth based timer is used to prioritise nodes when reserving a slot. This allows a node located in a deeper area to reserve a slot sooner than its above neighbouring nodes. The value of this timer at each node is given by

(2) $$T_{sch}=\left(\frac{2M_{Depth}}{M_{Depth}+N_{Depth}}\times T_{Delay}\right)-T_{Delay},$$

where $M_{Depth}$ is the depth of the network area and $N_{Depth}$ is a node depth in the network. $T_{Delay}$ is the length of the scheduling phase that is a predefined fixed value, set at the deployment time based on the application requirements. The value of $T_{Delay}$ depends also on the density of the nodes in an underwater area. It should be long enough to avoid collisions between neighbouring nodes, which are vertically very close to each other, during the scheduling phase. It is set to a small value for sparse and shallow scenarios and set to large values for deep and dense networks. Either way, the length of this phase is smaller than that of the third phase by a few order of magnitude. In order to synchronise all sensor nodes to begin and end at the same time, the length of the scheduling phase should be fixed.

As an example shown in Figure 2, the depth of nodes A, B, and C are assumed to be 500 m, 450 m, and 420 m, respectively. The network depth is 500 m and $T_{Delay}$ is set as 30 s. Using Equation (2), the scheduling time of node A, B, and C can be calculated as 0, 1.579, and 2.609 s, respectively. It shows that Equation (2) can prioritise sensor nodes based on their depth and the difference between their transmission times is long enough to avoid any overlapping.

A node before broadcasting its schedule packet, $S_{p}$, extracts the neighbouring nodes with lower depth than itself from $N_{t}$ and place them in depth priority list, DPL, in order of their depths. Thereafter, each node broadcasts its $S_{p}$ to its neighbouring nodes using depth-based timer. The $S_{p}$ includes the reserved sender slot and its DPL. Upon receiving a $S_{p}$, every node compares its $N_{t}$ with the received DPL to detect the hidden nodes, and also updates the reserved slots list based on the received $S_{p}.slot$.

This procedure is continuously executed by nodes in order of their depths and all nodes gradually reserve their own transmission slots while also informing their neighbouring nodes about that. Algorithm 1 shows how a node reserves a slot and provide other neighbouring nodes with some information including its reserved slot and a list of some of its neighbouring nodes. By the end of the scheduling phase, all sensor nodes are guaranteed to be scheduled. The scheduling phase is started by the nodes at the bottom of the water and ended by the nodes on the water surface. If the nodes on the surface are scheduled within $T_{Delay}$, it means that all other nodes located below are already scheduled. For example, if a sensor node is placed at the water surface with the depth 0, based on Equation (2), the node is scheduled at $T_{Delay}$, which is still within the duration of the scheduling phase.

**Algorithm 1** ED-MAC Scheduling1:**procedure** Schedule Packet2:    **if** depth-based timer is expired **then**3:        *S_p_*: a new schedule packet4:        *S_p_.slot* ← Slot-Selection (*N_t_*, *Reserved-slots*)5:        update *T_wake-up_* based on *S_p_.slot*6:        $S_{p}.D\;P\;L$ ← {*one-hop neighbours with lower depth ordered by their depth*}7:        *S_p_.ID* ← *N.ID*8:        **Broadcast**
*S_p_*9:    **end if**10:
**end procedure**
11:**procedure** Receive Schedule Packet (*S_p_*)12:    **if**
*S_p_* received **then**13:        update *N.Reserved-slots list*14:        **if**
*N.depth* < *S_p_.depth*
**then**15:            update *N_t_ by two-hop neighouring nodes*16:        **else**17:           update *T_wake-up_* based on *S_p_.slot*18:        **end if**19:    **end if**20:    **end procedure**

### 5.4. Normal Operational Phase

In this phase, nodes wake up and sleep periodically. They are awake in some slots and asleep during the remaining slots when there is no data transmission or reception. This phase is divided into a number of rounds and each round is consisted of a number of slots. Every slot also has a number of sub-slots. These slots are reserved by the nodes in the scheduling phase. The sub-slots are selected randomly to deal with the possibility of collisions between two nodes, if they have selected the same slot.

The length of each round, $T_{r}$, has a reverse relationship with offered traffic, $D_{rate}$, which is presented in terms of packet per second. The higher the offered traffic, the shorter the round time and, hence, the shorter sleeping time. The duration of each round is given by

(3) $$T_{r}=\frac{1}{D_{rate}}.$$

Each $T_{r}$ is divided into a number of slots, $N_{s}$. The number of slots per round is proportional to the maximum number of nodes in a neighbourhood, $N_{max}$. To exclude the possibility of concurrent data transmission from nodes located outside of one-hop neighbourhood and the node within the neighbourhood, the number of slots are doubled per round. We derive $N_{s}$ by using

(4) $$N_{s}=2\times N_{max},$$

where $N_{max}$ is the estimated maximum number of nodes in a particular one-hop neighbourhood. $N_{max}$ can be estimated based on the network topology, number of sensor nodes, and network dimensions and it can also be known to all nodes during the deployment time. The length of each slot, $T_{s}$, can be calculated using

(5) $$T_{s}=\frac{T_{r}}{N_{s}}.$$

**Figure 4:**
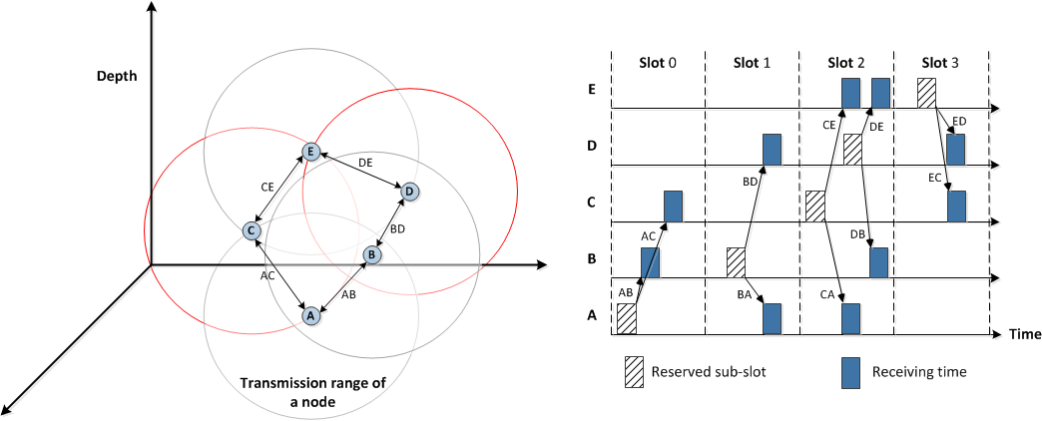
A specific scenario leading to a potential collision which has been addressed in ED-MAC.

Our proposed algorithm is almost a collision-free algorithm for most of scenarios. However, one slot might be reserved, under a very specific circumstances, by two hidden nodes, which both are neighbouring nodes of another node with lower depth. This specific scenario is illustrated in Figure 4. Node *A* has reserved slot $n,0\leq n\leq (N_{s}\;-\;1)$ and, consequently, slots $\left(n+1\right)\;mod\;\left(N_{s}\;-\;1\right)$ and $\left(n+2\right)\;mod\;\left(N_{s}\;-\;1\right)$ are reserved by its neighbouring nodes *B* and *C*, respectively. As node *D* is not aware of the slot number reserved by node *C*, it reserves the same slot, i.e., slot $\left(n+2\right)\;mod\;\left(N_{s}\;-\;1\right)$. Now, transmitting a packet by nodes *C* and *D* may result in a collision in node *E*.

To address this issue, the concept of sub-slot is introduced here. Each slot is divided into a number of equal size sub-slots. To transmit a packet, a node randomly selects one sub-slot out of $N_{s_{s}}$ available sub-slots to transmit its packet. The length of every sub-slot, $T_{s_{s}}$, is longer than a signal propagation delay to ensure that a packet is entirely received at the destination before starting of data transmission by another node. The length of each sub-slot, $T_{s_{s}}$, is given by

(6) $$T_{s_{s}}=T_{d}+T_{guard},$$

where $T_{guard}$ indicates the guard time, which is used to ensure that distinct transmissions do not interfere with one another and $T_{d}$ denotes the propagation delay of a transmitted packet which can also be calculated using

(7) $$T_{d}=\frac{R_{tr}}{U_{s}},$$

where $R_{tr}$ denotes the transmission range of a sensor node, and $U_{s}$ indicates the speed of sound in water. The number of sub-slots, $N_{s_{s}}$, can finally be calculated using the following equation:

(8) $$N_{s_{s}}=\frac{T_{s}}{T_{s_{s}}}.$$

**Figure 5:**
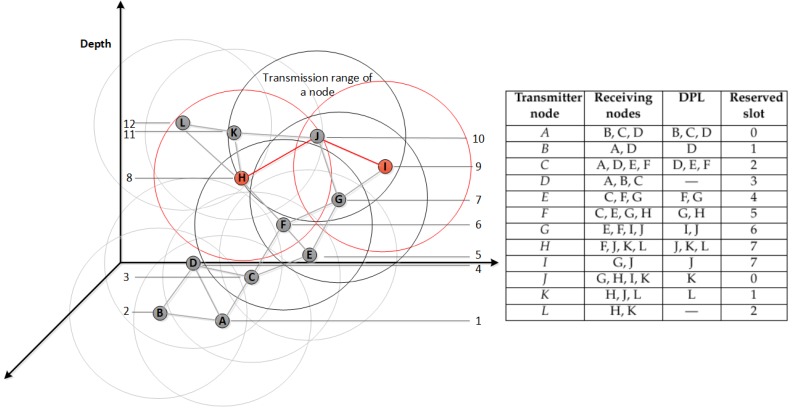
An example of depth-based scheduling in ED-MAC along with the scheduling results.

To show how our proposed MAC protocol provides a conflict-free scheduling, the simple network illustrated in Figure 5 is used. Once all sensor nodes, from *A* to *L*, exchange their one-hop neighbouring information, each one has its neighbours’ ID and depth. Hence, a deeper node, *A*, has a higher priority to reserve the first available slot time sooner than its lower depth neighbouring nodes *B*, *C*, and *D*. It also needs to know which slots are reserved by them, as one-hop neighbouring nodes, to schedule its wake up time.

After reserving a time slot and in order to address the hidden terminal problem, every node attaches an ordered, based on their depths, list of neighbouring nodes located at a lower depth than itself to the scheduling packet. This is to avoid any overlapping among two-hop neighbours. The scheduling results table, as shown on the right-hand side of Figure 5, shows the receiving nodes of each scheduling packet by each node along with the associated DPL and the reserved slot.

However, one rare case of collision might be occurred by two nodes located outside each others’ transmission ranges. Hence, one slot might be reserved by two nodes. This happens, as shown in Figure 5, when nodes *H* and *I* are both neighbouring nodes, with higher depth, of node *J* which are hidden from each others. Node *F* reserved slot *n*, $0\leq n\leq (N_{s}\;-\;1)$. Nodes *G* and *H* receive the scheduling packet of node *F* which includes its reserved slot and its DPL. Consequently, slots $\left(n+1\right)\;mod\;\left(N_{s}\;-\;1\right)$ and $\left(n+2\right)\;mod\;\left(N_{s}\;-\;1\right)$ are reserved by nodes *G* and *H*, respectively. This is mainly because both nodes are one-hop neighbours located in lower depth than node *F*. Node *I* knows the slot number reserved by *G*, but is not aware of the reserved slot by *H*, so it reserves the same slot $\left(n+2\right)\;mod\;\left(N_{s}\;-\;1\right)$. As a result, transmitting a packet by nodes *H* and *I* may lead to a collision at node *J*.

**Figure 6:**
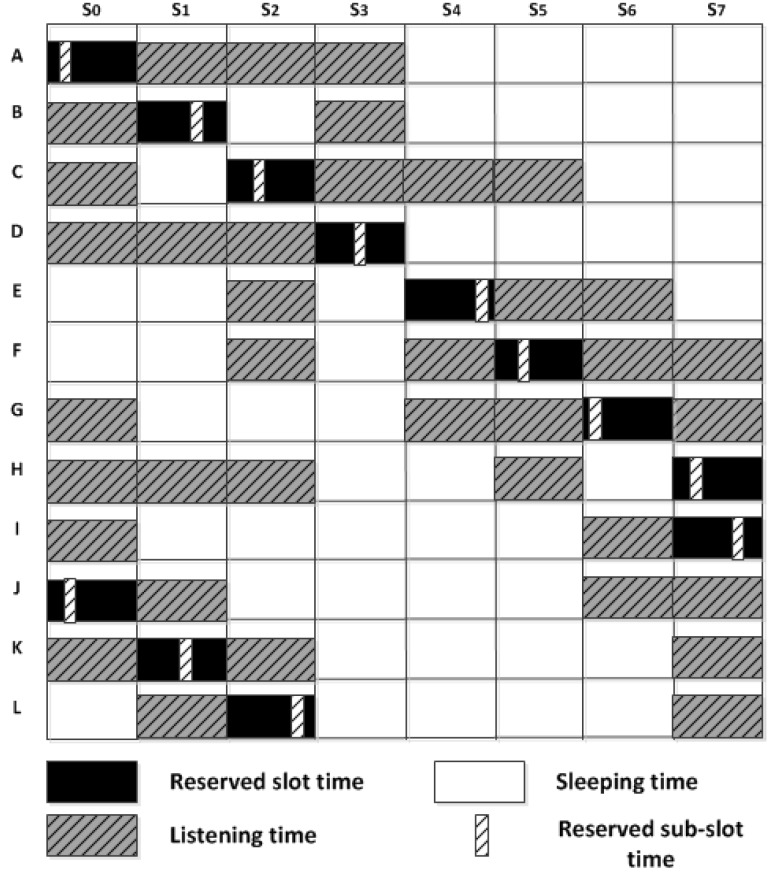
Network schedule between node *A* and node *L*.

To address this issue, the concept of sub-slots is introduced. In this case, both *H* and *I* nodes transmit their packets by reserving the same slot and then by randomly selecting different sub-slots out of number of available sub-slots, $N_{s_{s}}$, thereby node *J*, as a receiver, is able to collect both packets transmitted after each other without collision, as presented in Figure 6.

Moreover, Figure 6 illustrates how all the sensor nodes, in Figure 5, schedule their transmission period by reserving slot based on their depth priority: the greater the depth, the higher the priority to reserve a slot first. This figure also shows at what slot each node must wake-up to receive a packet from a neighbouring node according to the network topology given in Figure 5. In this case, the number of slots, $N_{s}$, is 8 by doubling the maximum one-hop neighbouring nodes, $N_{max}$, as shown in Equation (4). It should be noted that any two-hop neighbouring nodes or more can simultaneously be operated in the same slot, such as nodes *A* and *J*.

In the following example, we explain how a sensor node in the network topology given in Figure 5 assigns a slot based on its depth priority. Node *F* received the scheduling packets from nodes *C* and *E*, as one-hop neighbouring nodes located at a higher depth than itself, which include their reserved slots and DPLs. Node *C* reserves slot 2 because its DPL contains the one-hop neighbouring nodes located at a lower depth than itself such as nodes *D*, *E*, and *F*. Node *E* reserves slot 4 because it knows that node *D* is going to reserve slot 3 by the DPL of node *C*. By receiving both depth priority lists of nodes *C* and *E* and their reserved slots, the next available slot that can be used by node *F* is slot number 5, as depicted in Figure 6.

### 5.5. Handling Newcomers

In this sub-section, we introduce the concept of the newcomer sensor nodes and how ED-MAC protocol can deal with them. A newcomer sensor is a node that joins the network during the operational phase, which needs to be scheduled for transmitting and receiving data packets. This procedure can be made by letting the newcomers listening to their one-hop neighbouring nodes. The newcomer node can then select the first available slot and inform its neighbouring nodes by sending a control message. This message contains the available slot chosen by the newcomer node during the listening mode.

Due to an inability of nodes to hear from more than one-hop neighbouring nodes, a newcomer may select the same slot as the two-hop neighbours; however, the random selected sub-slots can be different to avoid collision. Therefore, the concept of the sub-slots is not only designed for a very specific collision scenario but also for handling newcomer nodes.

**Figure 7:**
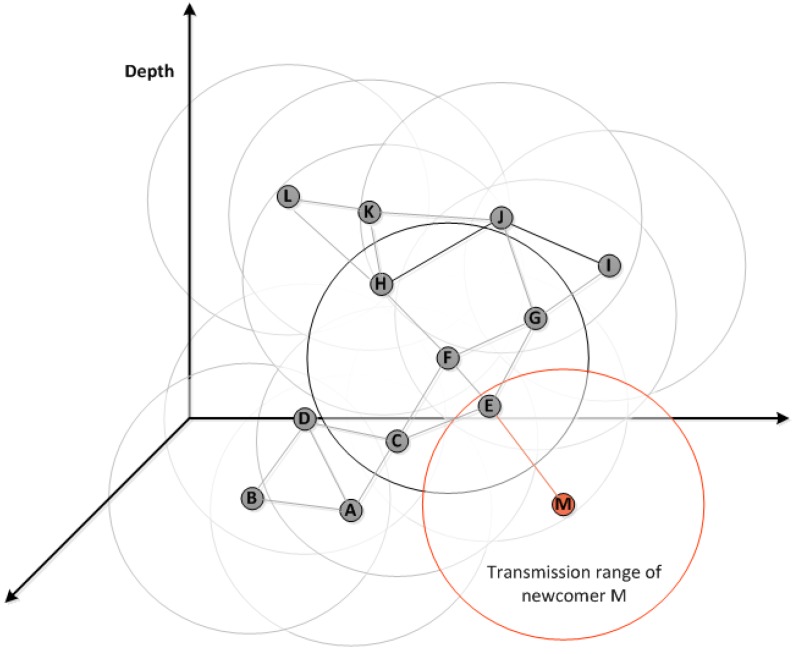
An example of how a newcomer, node *M*, joins the underwater network.

The topology in Figure 7 can be used to describe how the ED-MAC protocol handles newcomer sensor nodes in detail. In this figure, for instance, and by assuming that node *M* joins the network, where node *E* becomes its one-hop neighbouring node. Upon joining the network, it switches to listening mode until it hears from its one-hop neighbouring nodes. Thereafter, *M* broadcasts a *Hello* message back to its neighbourhoods to inform them about its transmission slot that selected during the listening duration. If this slot interferes with other neighbouring nodes of node *E*, which are considered as two-hop neighbours of node *M*, such that nodes *C*, *F* and *G*, the random selection of sub-slots can avoid the probability of collisions. For example, by assuming that node *M*, as a newcomer node, selects the same slot of node *C*, which is two hops away from each others and cannot be known by node *M* during the listening time. In this case, both *M* and *C* nodes use the same slot to transmit their packets to node *E* as a reception node. Consequently, transmitting a packet by nodes *C* and *M* may result in a collision at node *E*. Hence, *C* and *M* nodes select a random sub-slot out of those available to send their packets. In this way, nodes *M* and *C* can transmit their packets after each others by using two different sub-slots with no chance of collision.

**Figure 8:**
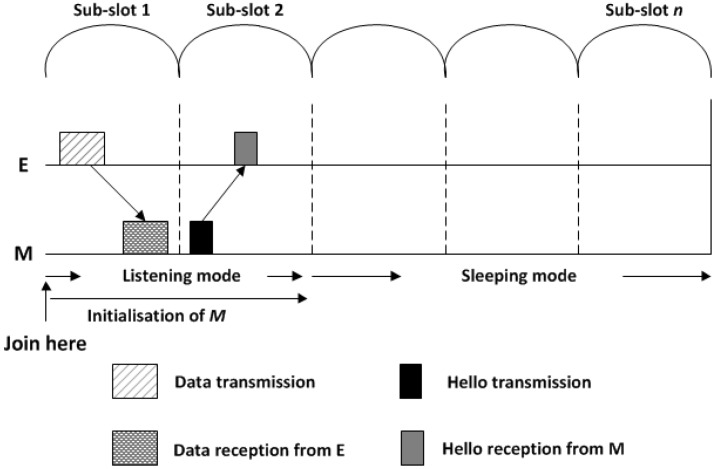
New node *M* joins the network.

Whenever a node transmits its packet, it will then be in a listening mode for the next sub-slot to receive a *Hello* message from newcomer sensor nodes, if there are any, as illustrated in Figure 8.

### 5.6. Offered Traffic Upper-Bound

The range of offered traffic, $D_{rate}$, used in Equation (3) should be carefully specified to be used in our system model. To find its upper-bound, the following equation must be satisfied:

(9) $$T_{s_{s}}\leq T_{s}.$$

It means that the slot length cannot be less than a sub-slot length, which is already calculated by Equations (5) and (6), respectively. By replacing $T_{s}$ with Equation (5), it can be presented as:

(10) $$T_{s_{s}}\leq \frac{T_{r}}{N_{s}}.$$

By replacing $T_{r}$ and $N_{s}$ using Equations (3) and (4), respectively, it can be extended as:

(11) $$T_{s_{s}}\leq \frac{1}{2D_{rate}\times N_{max}}.$$

Based on the above equation, the upper-bound for $D_{rate}$ is calculated as:

(12) $$D_{rate}\leq \frac{1}{2T_{s_{s}}\times N_{max}},$$

which shows that it depends on the sub-slot length and neighbouring node density. The sub-slot length, $T_{s_{s}}$, is a fixed value that is long enough to handle the consecutive receiving packets and $N_{max}$ is related to the node deployment model, number of nodes, and network topology.

In higher throughput, the length of round time is decreased based on Equation (3). The higher the offered traffic, the shorter round time and, hence, the shorter slot length based on Equation (5). The number of sub-slots is also decreased based on Equation (8). It means that the higher the offered traffic, the less sleeping time for each node, resulting in high throughput. In other words, when the traffic load is low, ED-MAC allows nodes to be in the sleeping mode for a long time to save more energy. However, when the traffic load is high, all nodes are active most of the time to send or receive data packets.

### 5.7. Number of Slots Analysis

ED-MAC can support broadcast transmissions in which any two nodes that are strictly more than two-hops away from each other can transmit simultaneously without interfering. ED-MAC is said to be collision free if and only if any two nodes, that are two-hop neighbours, do not have the same slot.

**Definition** **1:** *Let G=(V,E) be an undirected graph representing the network topology. V includes all the nodes in the network while E represents all edges between two neighbouring nodes.*

**Definition** **2:** *The number of h-hop neighbours of a vertex v, denoted by $d_{h}\left(v\right)$, is called the h-hop degree of v. The maximum h-hop degree vertex in a graph G, $\Delta_{h}\left(G\right)$, defines the h-hop graph degree.*

**Theorem** **1:** *If $\Delta_{2}\left(G\right)=d$, all nodes in G can be scheduled with d+1 slots without having slot overlap between any two-hop neighbouring pairs.*

**Proof.**  Since each node has at most *d* two-hop neighbours, there is always at least one free slot in the range {1,…,d+1}.  ☐

**Figure 9:**
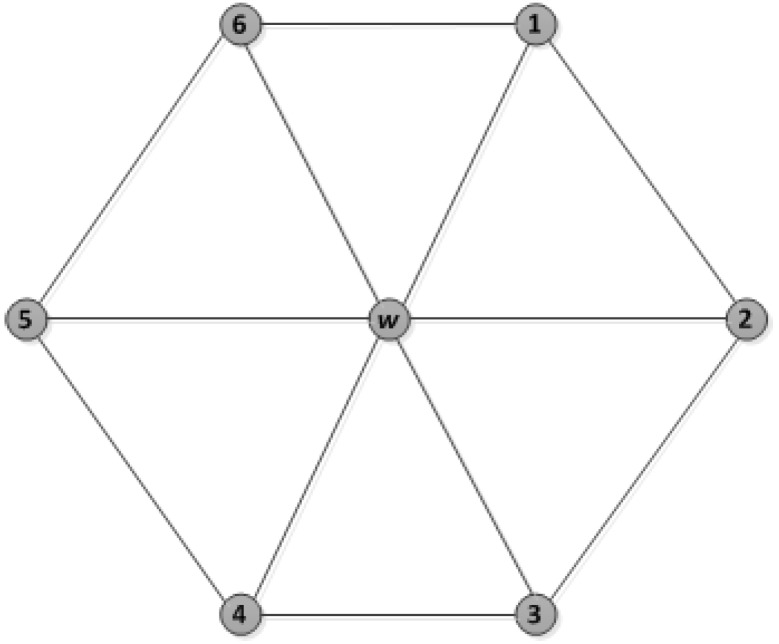
Network design.

**Figure 10:**
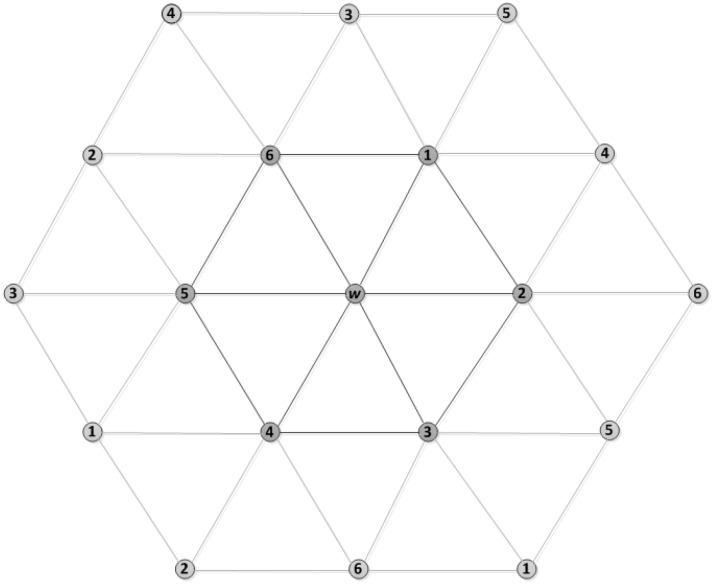
Network layout with two-hop neighbours of *w*.

For the network design shown in Figure 9, in this figure, node *w* has a number of *N* neighbours (in this case *N* = 6, numbered 1 through 6 in the figure). Each of them is connected through *w* as a neighbourhood. Thus, the upper bound can be handled by *w* = {d+1}, where *d* = 6. Since some of these have *N* neighbours which are two-hop neighbours of *w* as shown in Figure 10, this can also be handled by assigning two-hop distance slots.

It should be noticed that d+1 is only an upper bound because it might be possible to schedule nodes with fewer slots.

## 6. Performance Evaluation

In this section, we first describe and compare ED-MAC with UWAN-MAC [17] and T-Lohi [41] protocols qualitatively. We then discuss the implementation of our protocol, ED-MAC, in the Aqua-Sim underwater simulation [49]. We next specify the metrics used in our performance study to evaluate the performance of ED-MAC protocol against those of two protocols from the same category recently reported in the literature. We finally present and analyse the simulation results.

### 6.1. Qualitative Comparison

Both ED-MAC and T-Lohi are deterministic reservation-based MAC protocols. The main difference between the two protocols is that T-Lohi schedules its transmission data by using the presence of a contention phase, and it uses tones at the beginning of each round to reserve the channel. In ED-MAC, after exchanging the neighbouring nodes’ information, every sensor node reserves its own slot for its data transmission by using a depth-based timer within only a short scheduling phase compared to the operational phase.

During the operating phase, with the dismantling of the slots and sub-slots, ED-MAC acts like UWAN-MAC, with the main difference being that it has a deterministic behaviour. UWAN-MAC has a stochastic behaviour in which the sender nodes select random times to send their packets, thereby increasing the number of collisions; however, ED-MAC uniformly distributes the sending times over a given round to make better use of the channel.

T-Lohi and UWAN-MAC are performed in two different manners. The main difference between these two MAC protocols is the presence of the contention phase in T-Lohi (which is absent in UWAN-MAC). Moreover, nodes in UWAN-MAC enter a back-off state whenever activity is overheard on the channel. While in T-Lohi, the back-off is only necessary to drive the contention phase, so that a single transmitter is granted channel access.

### 6.2. Implementation

We implement ED-MAC in Aqua-Sim, an NS-2 based simulator for underwater sensor networks. Unless specified otherwise, we use the following values are used for various parameters in our simulation, which is widely used in the literature [6,50]. The power consumption on transmission mode is two watts, the power consumption on receive mode is 0.75 Watts, and the power consumption on sleep mode is 8 mW. The data packet size is set to 2000 bits and the control packet size is set to 100 bits. The bit rate is 10 Kbps and the maximum transmission range is 100 meters. We set the simulation time to be 3600 s. Our simulation results are presented by varying two parameters: offered traffic and number of nodes to study the load effects and network scalability in each protocol. In the first parameter, all nodes are distributed in a 10,000 m^2^ × 200 m area. We deploy 10 nodes into the network with varying offered traffic in this area. In the second parameter, however, all nodes are distributed within an area of 62,500 m^2^ × 500 m, while keeping the offered traffic fixed to 0.25 packets per second and increasing the number of nodes until 100 nodes in this narrow area. In either way, all sensor nodes, located between a sink and anchored nodes, can move horizontally within the speed of 2 m/s by following a Random Walk 2D mobility model. This movement can only be in an *X*-*Y* plane, which is mostly used in an underwater environment. In our simulation setup, we consider $T_{b}$ and $T_{Delay}$ as 30 s each. The simulation parameters are summarised in Table 2.

### 6.3. Performance Metrics

We define four metrics to compare the performance of ED-MAC protocol with those of UWAN-MAC and T-Lohi protocols, namely, successful delivery ratio, throughput, energy consumption, and fairness index. The successful delivery ratio is defined as the ratio of the number of packets received successfully to the total number of packets generated in the network. The network throughput refers to the total amount of data successfully transmitted by the network within a given period of time. The energy consumption is obtained by dividing the overall energy consumption in the network by the successfully delivered data packets, which is measured in joules per packet.

The fairness index is a key performance metric when studying MAC protocols to measure the equality of users to access the media. To evaluate the fairness of underlying protocols, we adopt the Jain’s Fairness Index [51]:

(13) $$\mathrm{Fairness}\;\mathrm{Index}=\frac{{\left(\sum x_{i}\right)}^{2}}{\left(n.\sum x_{i}^{2}\right)},$$

where $x_{i}$ denotes the throughput of node *i*, 1<=i<=n, and *n* denotes the number of sensor nodes in the network. This value ranges between 0 and 1 to be used suitably as a measure of fairness. The fairness increases when this value becomes closer to 1. As the disparity increases, fairness decreases and the fairness index becomes closer to 0. Accessing the media is totally fair when the fairness index equals 1.

### 6.4. Simulation Results

The performance of ED-MAC is compared with those of UWAN-MAC and T-Lohi through simulations. For each scenario, the results are averaged over 50 runs, with a randomly generated topology in each run. The simulation results within two parameters studies the traffic effect and network scalability in every protocol and also shows how these three MAC protocols perform in practical underwater networks.

**Figure 11:**
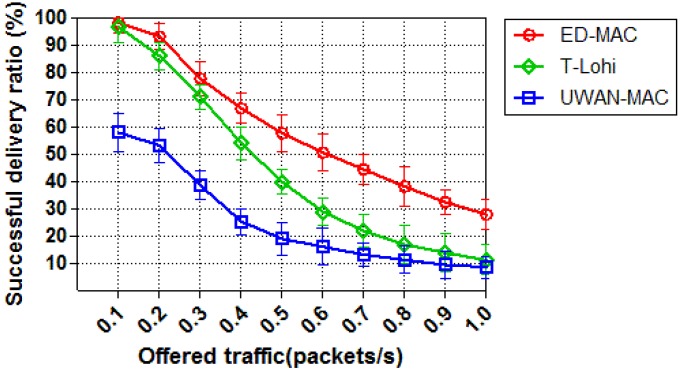
Successful delivery ratio vs. offered traffic.

In the first set of simulations, we compare the successful delivery ratio of all three protocols by varying offered traffic while the number of node equals to 10. Figure 11 shows the successful delivery ratio of each protocol as a function of offered traffic. The successful delivery ratio of ED-MAC outperforms that of other two MAC protocols, T-Lohi and UWAN-MAC. This is because ED-MAC has the ability to address the hidden node and the spatio-temporal uncertainty problems which cannot be detected by UWAN-MAC protocol resulting in more packet loss and collisions. At a low traffic load, the successful delivery ratio of ED-MAC and T-Lohi almost achieves 100%, while UWAN-MAC only reaches 60%. This is due to the inefficient scheduling of UWAN-MAC, which causes considerably more collisions and retransmissions, where ED-MAC and T-Lohi exploit space-time uncertainty and high latency to avoid collisions and retransmissions. When the traffic is further increased, however, T-Lohi shows a fast degradation of the successful delivery ratio due to an increasing number of collisions of tone packets. The performance of ED-MAC in this simulation is far better than that of T-Lohi and UWAN-MAC, as ED-MAC employs a priority approach based on the depth of each sensor node to schedule the transmission and also to avoid data collisions. ED-MAC also allows more communications in one slot, by using temporal and spatial reuse, which significantly increases the acoustic channel utilisation for underwater networks.

**Figure 12:**
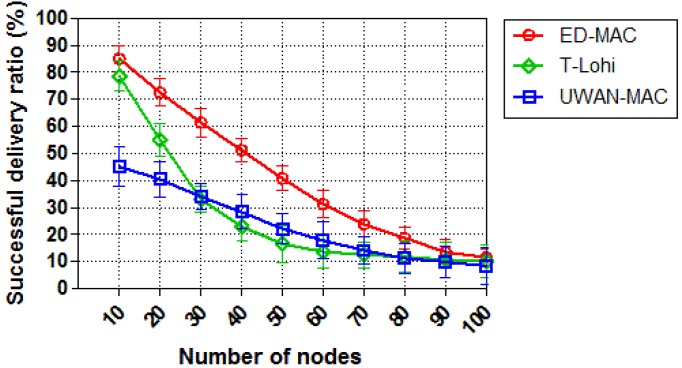
Successful delivery ratio vs. node density.

We then study the network scalability of each protocol by increasing the sensor nodes and keeping the offered traffic constant (with 0.25 packets/s). As can be seen from Figure 12, the successful delivery ratio of all three protocols is inversely proportional to the number of nodes. With 10 nodes, ED-MAC reaches 88% compared to 78% and 45% for T-Lohi and UWAN-MAC, respectively. This is due to the specific benefits of ED-MAC, such as high scalable scheduling; consequently, it can handle more packets than either T-Lohi or UWAN-MAC. When the number of nodes is increased, T-Lohi’s successful delivery ratio rapidly reduces due to an increasing number of collisions of tone packets, thus resulting in incorrect reservations. The impact of increasing the number of nodes on the successful delivery ratio in T-Lohi is more profound. Still more interesting, however, is that UWAN-MAC only achieved 45% of the successful delivery ratio (within 10 nodes), which is much lower than the successful delivery ratio of either ED-MAC or T-Lohi. This is because UWAN-MAC is designed to only use one control packet across the network.

**Figure 13:**
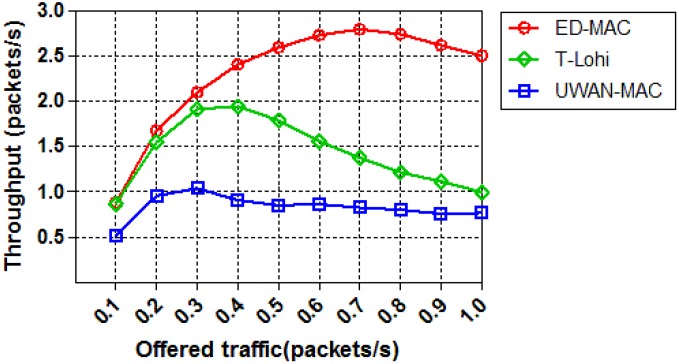
Throughput vs. offered traffic.

The second set of simulations studies how the load affects the network throughput of those three protocols with only 10 sensor nodes. As shown in Figure 13, when the offered traffic increases, the network throughput increases correspondingly and finally reaches the saturation status. ED-MAC achieves higher network throughput than that of UWAN-MAC and T-Lohi when their traffic loads are the same. This is because ED-MAC uses a depth-based timer that allows each node to prioritise slot reservation depending on the node depth; the greater the depth, the higher the priority to reserve a slot first. In both UWAN-MAC and T-Lohi, the spatial-temporal uncertainty problem is not considered and therefore their network throughput are lower than that of ED-MAC. Specifically, ED-MAC improves 37.8% of the network throughput than that of T-Lohi and 63.9% of the network throughput than that of UWAN-MAC on average.

**Figure 14:**
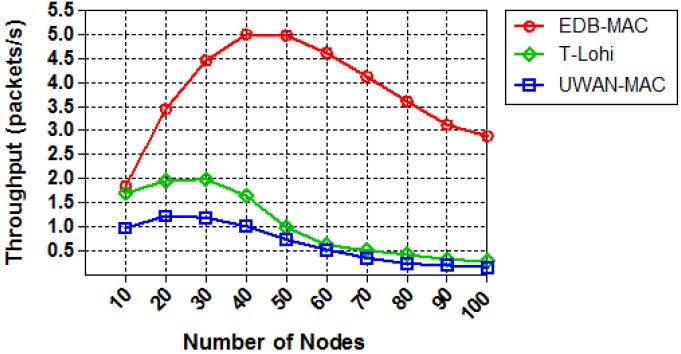
Throughput vs. node density.

We next compare the throughput of those protocols by increasing the node density while the offered traffic is kept fixed to 0.25 packets per second. Figure 14 shows the throughput of the three protocols as a proportional to the number of nodes. As the number of nodes increases, the network throughput increases correspondingly and eventually reaches a saturation point. ED-MAC reaches higher network throughput and performs better than other two MAC protocols in the same circumstances. This is due to its specific benefits from a high scalable scheduling to handle more data packets than T-Lohi and UWAN-MAC. Moreover, the curve of ED-MAC exhibits significantly increased by achieving five packets per second within 50 nodes, and then a slower decline compared with that of T-Lohi and UWAN-MAC, which proves the effectiveness of the proposed protocol. Thus, the scalability of handling more packets in ED-MAC outperforms that in T-Lohi and UWAN-MAC.

**Figure 15:**
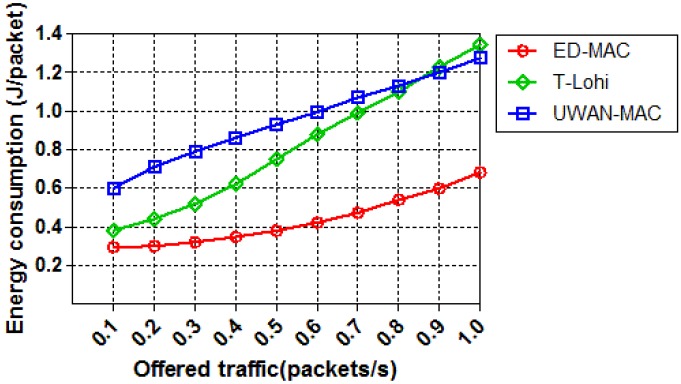
Energy consumption vs. offered traffic.

The third set of simulations evaluates how the offered traffic affects the energy consumption of the three different MAC protocols with 10 nodes. Figure 15 demonstrates that ED-MAC is much more energy efficient in this simulation than T-Lohi and UWAN-MAC. At a low traffic load, ED-MAC is still more energy efficient than other protocols due to its lower receiving overhead and less idle overhead per data packet. Another reason for ED-MAC’s superior efficiency is that it has a compatible schedule based on its depth criteria, which helps to avoid the possibility of collisions. As the traffic load increases, T-Lohi shows a rapid rise of the energy consumption per packet due to an increasing number of collisions of control packets resulting in incorrect reservations. It is more interesting to observe that when the traffic load increases, the energy consumption of ED-MAC also slightly increases, although it still has a higher network throughput than T-Lohi and UWAN-MAC protocols.

**Figure 16:**
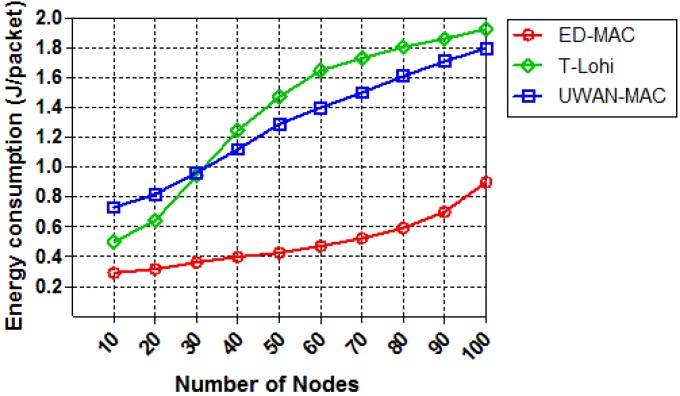
Energy consumption vs. node density.

We also investigate the effect of increasing the node density in each protocol in terms of energy consumption while the offered traffic is constant. As shown in Figure 16, the energy consumption of the three protocols is drawn as a function of the number of nodes. As the number of nodes increases, the energy consumption increases correspondingly. This is because, when the number of nodes increases, more nodes are involved; therefore, there is more intensive competition to access the channel. ED-MAC consumes the lowest energy among the two other protocols because it adopts energy conservation measures by considering hidden terminal and spatial-temporal uncertainty problems. As the node density increases, T-Lohi consumes significantly more energy per packet due to an increasing number of collisions. Specifically, ED-MAC consumes on average only 61.5% of the energy consumption of UWAN-MAC and 63.8% of the energy consumption of T-Lohi. T-Lohi consumes the highest energy among other protocols, although it conserves energy by employing a wake-up tone receiver that allows very low-power listening for wake-up tones.

**Figure 17:**
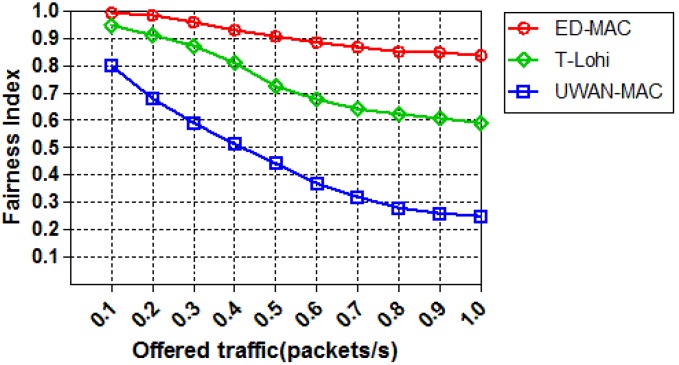
Fairness vs. offered traffic.

In the last set of simulations, we study the effect of increasing the offered traffic on the fairness index among those three protocols. Figure 17 shows the result of an experimental setup consisting of ten sensor nodes that are run for an hour to strenuously test the fairness of the three protocols. First, it is observed that the ED-MAC protocol exhibits a high fairness index of almost 98%, which then slightly decreases over all the traffic loads. In comparison, the fairness index of T-Lohi and UWAN-MAC (within 0.1 offered traffic) achieves nearly 94% and 80%, respectively. When the offered traffic further increases, the fairness index of the latter protocols significantly decreases. This is mainly because of an extensive competition between nodes to access the channel. ED-MAC protocol achieves higher fairness of transmission and throughput than other protocols due to the channel reservation, which is based on the depth criteria.

**Figure 18:**
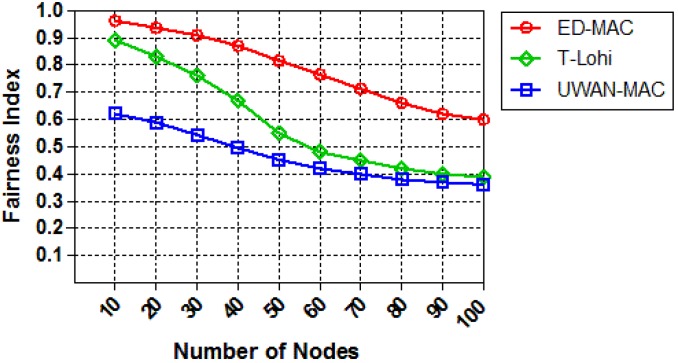
Fairness vs. node density.

We finally evaluate the fairness index of each protocol by increasing the number of sensor nodes while keeping the load constant at (0.25 packets/s). As illustrated in Figure 18, when the number of nodes increases, the fairness index of all protocols declines significantly. This is because the network congestion reduces the fairness in all three protocols. Due to the large delays in the underwater acoustic network, the distance between nodes becomes a key factor in the competitive channel. In contrast, the fairness index of ED-MAC (within 10 nodes) is higher than both T-Lohi and UWAN-MAC by approximately 8% and 35%, respectively. This is because the latter protocols are highly affected by long propagation delays, which cannot avoid the spatial-temporal uncertainty problem, leading to increasing the number of unfairness transmissions. Moreover, UWAN-MAC involves unknown propagation delays, which affects its fairness compared to other two protocols.

## 7. Conclusions

Designing a MAC protocol for underwater networks with acoustic communication is affected by many factors, the most important of which are long propagation delay, low bandwidth, and energy constraints. In this paper, we have proposed an efficient reservation-based distributed MAC protocol (ED-MAC) for underwater sensor networks. ED-MAC uses a duty cycle mechanism that can reduce energy consumption as well as improve throughput and fairness by handling the traffic contention effectively. The spatial-temporal uncertainty as well as the hidden node problem have also been addressed. Using an extensive simulation study, the performance of ED-MAC has been compared against those of two other protocols from the same category recently reported in the literature. Simulation results have shown that ED-MAC performs well in terms of the successful delivery ratio, throughput, energy consumption, and fairness index with varying offered traffic and number of nodes. In terms of the network scalability, ED-MAC highly outperforms those two other compared protocols. As future work, we plan to improve the energy efficiency and reliability by applying the knowledge of depth (layer) at each node. In this way, we are able to vertically and horizontally prevent any possibility of collisions between sensors while the scheduling phase is not based on the deep message exchange and the concept of timer. We also plan to consider the variable traffic load during the operational phase. In this case, the whole setup process is not required to be repeated.

## Figures and Tables

**Table 1:** Notations.

Terms	Definition
$B_{p}$	Beacon packet
DPL	Depth Priority List
$D_{rate}$	Offered traffic (packets/s)
$M_{Depth}$	The depth of the network (m)
N	underwater sensor node
$N_{Depth}$	A node depth in the network (m)
N.ID	Node’s ID
$N_{max}$	Maximum number of nodes per neighbourhood
$N_{s}$	Number of slots
$N_{s_{s}}$	Number of sub-slots
$N_{t}$	Neighbouring table
$R_{tr}$	Transmission range (m)
$S_{p}$	Schedule packet
$T_{b}$	Predefined fixed value for the initial phase (s)
$T_{d}$	Propagation delay (s)
$T_{Delay}$	Predefined maximum delay (s)
$T_{guard}$	Guard time (s)
$T_{r}$	Length of each round (s)
$T_{s}$	Length of each slot (s)
$T_{s_{s}}$	Length of each sub-slot (s)
$T_{sch}$	Scheduling timer of each node (s)
*T_wake-up_*	The wake-up times of a node (s)
$U_{s}$	Speed of sound in water (m/s)

**Table 2:** Simulation parameters.

Parameter	Value
Transmission power	2 Watts
Receiver power	0.75 Watts
Idle power	8 mW
Maximum transmission rage	100 m
Bandwidth	10 Kb/s
Acoustic propagation speed	1500 m/s
Offered traffic	0.1–1.0 packet/s
Node Number	10–100 sensor nodes
Deployment region	10,000 m^2^ × 200 m
	62,500 m^2^ × 500 m
Movement model	RandomWalk 2D mobility model
Movement speed of nodes	2 m/s, change movement
	direction every 2 s
Running rounds	50
Control packet size	100 bits
Data packet size	2000 bits
Length of the initial phase	30 s
Length of the scheduling phase	30 s
Simulation time of one round	3600 s
